# Research advances on the gut-kidney axis theory in sepsis-associated acute kidney injury

**DOI:** 10.3389/fphar.2026.1878375

**Published:** 2026-08-18

**Authors:** Min Li, Yu-Long He, Ling Chen, Peng-Zhong Fang, Fei Feng, Tian-Kui Shuai, Li-Ping Liu

**Affiliations:** Emergency Intensive Care Unit, First Hospital of Lanzhou University, Lanzhou, Gansu, China

**Keywords:** acute kidney injury, gut-kidney axis, intervention, mechanism, sepsis

## Abstract

Sepsis-associated acute kidney injury (SA-AKI) is a common, high-mortality complication in critically ill patients, necessitating breakthroughs in prevention and treatment strategies. The “gut-kidney axis” theory offers a novel perspective. This review delves into the vicious cycle triggered by intestinal barrier disruption and dysbiosis in SA-AKI: pathogenic translocation, immune cell infiltration, and imbalances in nephrotoxic metabolites collectively drive renal inflammatory storms. Building upon these mechanisms, we systematically evaluate novel therapeutic approaches centred on microecological modulation, targeted anti-inflammatory immunotherapy, traditional Chinese medicine interventions, and vagus nerve electrical stimulation. These strategies aim to pioneer new pathways for the precise prevention and treatment of SA-AKI.

## 1 Frontiers and research background

### 1.1 Epidemiology

Sepsis refers to life-threatening organ dysfunction caused by a dysregulated host response to infection (Evans et al., 2021). Globally, severe infections and sepsis represent the most common causes of acute kidney injury (AKI), with reported incidence rates showing significant variation, this is primarily due to differences in the selection of study populations, geographical regions, ICU settings, and diagnostic criteria for sepsis and AKI. A systematic review has shown that the overall incidence of sepsis-associated acute kidney injury (SA-AKI) among ICU patients with sepsis is approximately 40 percent; however, when studies are restricted to standardised definitions such as RIFLE, AKIN or KDIGO, this proportion rises to approximately 52 percent, with reported ranges in individual studies varying widely from 14 percent to 87 percent (Zarbock et al., 2023b; Donaldson et al., 2024). Following the adoption of the ADQI 28 consensus definition, studies in adult ICUs have consistently shown that SA-AKI affects nearly half of all patients with sepsis and accounts for approximately one-sixth of all ICU admissions, with the majority of cases manifesting in the early stages of sepsis (Takeuchi et al., 2025). When considering specific clinical populations, this burden may be even higher—for example, the incidence of SA-AKI reached 62.3% in a South Korean ICU sepsis cohort, global ICU audit data indicate that 68 percent of patients admitted to the ICU with sepsis have concomitant AKI, with the highest prevalence observed in patients with septic shock; whilst in a Beijing-wide cohort of hospitalised sepsis patients, the overall incidence still reached 48.1 percent, suggesting that non-ICU in-hospital populations also bear a significant burden (Peters et al., 2018; Liu et al., 2020; Wang et al., 2021; Song et al., 2024). Beyond differences at the population level, the risk of SA-AKI is also significantly stratified by baseline characteristics at the individual host level; advanced age is an independent risk factor for SA-AKI (Zhou et al., 2019), furthermore, patients with comorbidities such as diabetes, chronic liver disease, underlying heart disease, coagulopathy and a high APACHE II score exhibit a significantly increased incidence of SA-AKI (Liu P.et al., 2023). Overall, SA-AKI portends a poorer prognosis than the condition alone, leading to prolonged hospital stays, increased mortality, higher rates of long-term disability, and diminished quality of life (Balkrishna et al., 2023; Zarbock et al., 2023a). Its pathophysiological mechanisms are complex and multifaceted, involving multiple factors including systemic and local renal inflammatory responses, alterations in microcirculation and endothelial function, intrarenal shunting, complement system activation, renin-angiotensin-aldosterone system dysregulation, mitochondrial dysfunction, and metabolic reprogramming (Zarbock et al., 2023a). Despite significant advances in the detection and management of SA-AKI (Zarbock et al., 2023a),the substantial health burden it imposes as a critical illness remains undeniable. To mitigate its short- and long-term consequences, further investigation is warranted.

### 1.2 The scientific basis and research necessity of the gut-kidney axis theory

Traditional concepts have regarded the gut and kidneys as relatively independent organs in function. However, recent research has made a groundbreaking discovery: under pathological conditions, a close bidirectional communication pathway exists between the two, known as the “gut-kidney axis”. In 2011, Meijers and Evenepoel systematically elaborated upon this theory, noting that in chronic kidney disease (CKD), declining renal function and gut microbiota dysbiosis form a vicious cycle: reduced renal clearance of uraemic toxins (such as indole-3-sulphate) leads to their accumulation in the gut, triggering microbial dysbiosis. This dysbiosis further enhances toxin production and increases intestinal permeability, allowing toxins to enter the bloodstream. By inducing systemic inflammation and oxidative stress, this ultimately accelerates renal deterioration (Meijers and Evenepoel, 2011). This theory provides a crucial perspective for understanding multiple organ dysfunction in sepsis. Under the acute onslaught of sepsis, the gut microbiota—as a vital regulator of immunity and metabolism—interacts with the kidneys in a dynamic and complex process that remains incompletely understood (Xu et al., 2022). Systematically investigating the gut-kidney collaborative mechanisms in sepsis, not only deepens our understanding of the disease’s fundamental nature, but also holds promise for identifying novel therapeutic targets and insights for developing treatment strategies.

Consequently, this review aims to re-examine the mechanisms underlying the gut-kidney axis in SA-AKI and to systematically synthesise key research advances in this field in recent years. Unlike previous reviews, which primarily focused on the inflammatory response, alterations in the gut microbiota, or individual regulatory mechanisms of the gut-kidney axis in SA-AKI, this paper further emphasises the dynamic interrelationships between gut microbiota dysbiosis, disruption of the intestinal barrier, immune regulation, metabolic abnormalities and damage to distant organs, thereby establishing an integrated framework spanning from disease phenotypes to molecular mechanisms, and on to diagnostic biomarkers and therapeutic strategies.

At the mechanistic level, this paper systematically elucidates how the gut-kidney axis contributes to the onset and progression of SA-AKI by integrating recent basic research, preclinical models and clinical evidence, and further explores how these mechanistic insights drive the development of novel biomarkers and the formulation of targeted intervention strategies. Furthermore, this paper provides a comprehensive analysis of traditional diagnostic indicators, novel biomarkers, multi-omics technologies and precision treatment strategies, with the aim of advancing SA-AKI research from descriptive correlations towards mechanism-driven and clinically translational approaches, thereby providing a new theoretical basis for future precision diagnosis and treatment based on the gut-kidney axis.

## 2 Pathological mechanisms by which an imbalance in the gut-kidney axis mediates the onset and progression of SA-AKI

**FIGURE 1:**
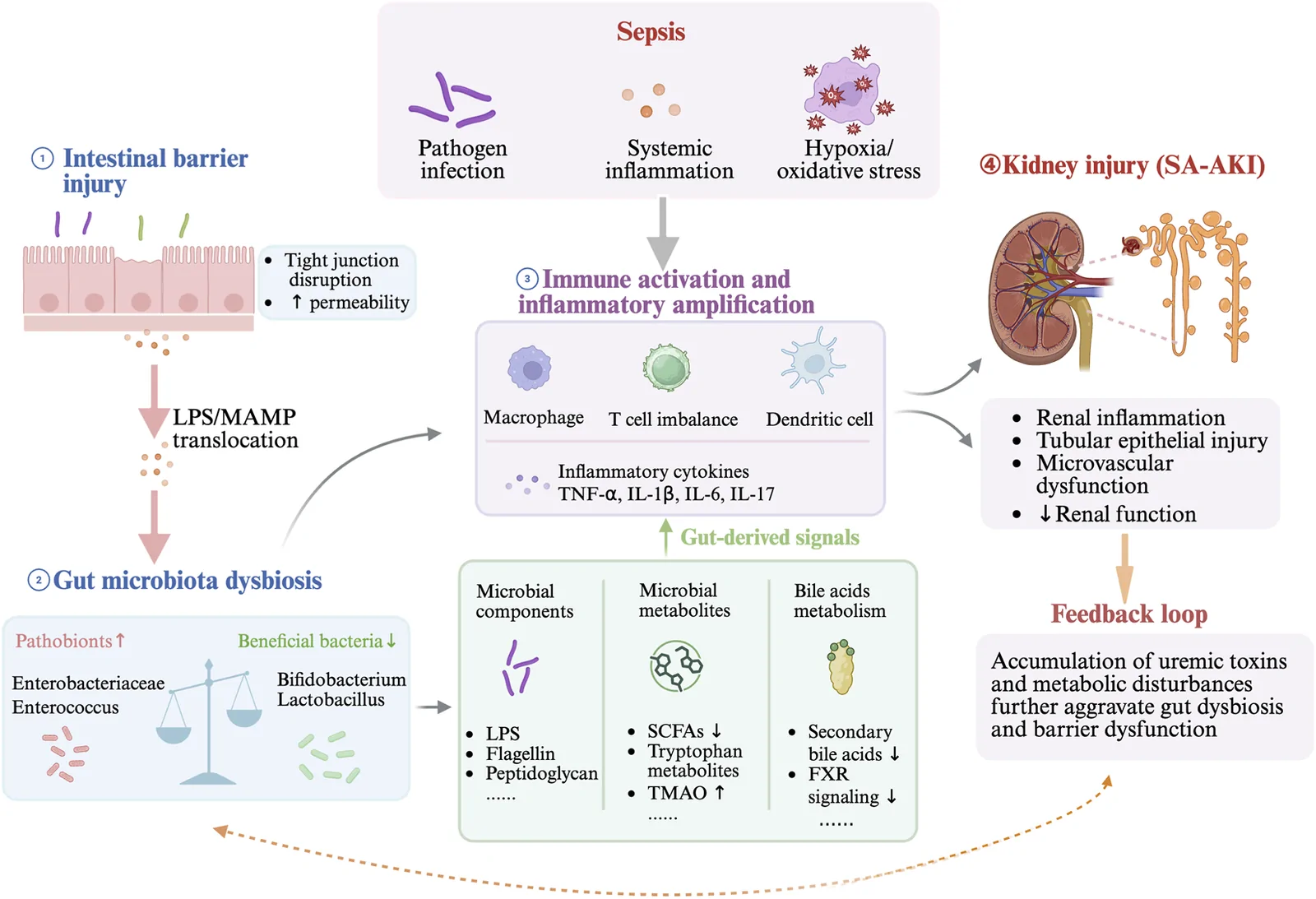
Sepsis triggers intestinal barrier disruption and gut microbiota dysbiosis, leading to the release of microbial components and metabolites into circulation. These gut-derived signals amplify systemic immune activation and inflammatory responses, resulting in renal inflammation, tubular injury, and microvascular dysfunction. Meanwhile, kidney dysfunction promotes uremic toxin accumulation and metabolic disturbances, further aggravating intestinal dysbiosis and barrier impairment, forming a vicious gut–kidney feedback loop.

The onset and progression of SA-AKI are not driven solely by local renal inflammatory responses or haemodynamic abnormalities, but are continuously regulated by interactions within the gut-kidney axis. In the early stages of sepsis, systemic inflammatory responses, tissue ischaemia and hypoxia, and oxidative stress can impair intestinal barrier function, facilitating the translocation of bacteria and microbe-associated molecular patterns (MAMPs) into the circulation, thereby further activating the inflammatory response. Subsequently, dysbiosis of the gut microbiota can accelerate the progression of SA-AKI by altering microbial metabolic functions, disrupting immune homeostasis and promoting inflammation-mediated renal injury. As renal function deteriorates, impaired clearance of metabolic waste products and uremic toxins exacerbates gut microecological disruption and barrier damage, ultimately forming a vicious cycle of ‘intestinal injury—amplified inflammation—renal dysfunction—further intestinal injury’. Consequently, intestinal barrier disruption, dysbiosis, immune dysregulation and metabolic abnormalities are not strictly sequential, independent stages, but rather a continuous pathological process in which these factors mutually reinforce one another and are dynamically intertwined throughout the course of sepsis (Figure 1).

### 2.1 Sepsis-induced intestinal barrier damage and the initiation of renal inflammation

In the early stages of the onset and progression of sepsis, disruption of the intestinal barrier is considered a key factor in triggering abnormalities in the gut-kidney axis. As one of the body’s largest immune and metabolic barriers, the intestine is susceptible to inflammation, ischaemia, hypoxia and oxidative stress during sepsis, and contributes to the amplification of systemic inflammation. The literature consistently indicates that there is a decrease in the expression of tight junction proteins in the intestinal epithelium and an increase in the opening of paracellular pathways; intestinal epithelial cells undergo apoptosis and necrosis under the stimulation of ischaemia, hypoxia and inflammatory cytokines, leading to a breach in the integrity of the mucosal barrier and increased intestinal permeability. Consequently, MAMPs are released into the circulation, leading to endotoxemia. This, in turn, activates pattern recognition receptors (primarily TLR4, whose key co-receptor is CD14) on systemic immune cells (such as macrophages), inducing the massive release of pro-inflammatory cytokines; this process is closely associated with renal injury ((Turner, 2009; Chen et al., 2017; McMullan et al., 2024). In animal models of sepsis, studies have directly demonstrated that displaced intestinal LPS activates renal resident cells and infiltrating immune cells via the CD14-TLR4-NF-κB signalling pathway, leading to the release of substantial pro-inflammatory factors (such as TNF-α, IL-1β, IL-6) that directly damage glomerular and tubular epithelial cells (Zhang et al., 2025).

Furthermore, abnormalities in bile acid metabolism mediated by the gut microbiota may further influence inflammasome activation and immune homeostasis, providing another potential regulatory mechanism for sepsis-associated abnormalities in the gut-kidney axis (Alimov et al., 2019).

Consequently, sepsis-induced disruption of the intestinal barrier not only leads to the translocation of bacteria and their associated metabolites into the intestinal lumen, but also serves as a crucial link between local intestinal damage and distal renal inflammation. However, as the disease progresses, barrier disruption alone is insufficient to explain the sustained amplification and long-term maintenance of the inflammatory response; dysbiosis of the gut microbiota and the resulting abnormalities in immune regulation further drive the disruption of the gut–kidney axis.

### 2.2 Inflammation and abnormal immune regulation driven by an imbalance in the gut microbiome

In a healthy state, the gut microbiota maintains a dynamic equilibrium, protecting the host through metabolic products, immune regulation, and competitive exclusion. Sepsis may induce dysbiosis of the gut microbiota, characterised by a reduction in beneficial bacteria (such as Bifidobacterium and *Lactobacillus*) and overgrowth of opportunistic pathogens including Enterobacteriaceae (Zhang et al., 2022). Consequently, the dysbiosis caused by sepsis is manifested not only by changes in microbial composition, more importantly, by alterations in microbial metabolic functions and the host’s ability to regulate the immune response, thereby further affecting the stability of the intestinal barrier and the inflammatory state of the kidneys.

Short-chain fatty acids (SCFAs) serve as pivotal functional molecules produced by gut microbiota metabolism, acting as important regulators of intestinal homeostasis. In various models of intestinal diseases, SCFAs have been shown to maintain intestinal barrier integrity through a variety of mechanisms, including promoting mucus secretion, strengthening tight junctions between epithelial cells, inhibiting pyroptosis and improving microcirculation (Huang et al., 2023; Li et al., 2023; Lou et al., 2023). Concurrently, SCFAs are also involved in systemic and renal immune regulation. In animal models of acute or chronic kidney disease, SCFAs can reduce the levels of pro-inflammatory factors (TNF-α, IL-1β, IL-17A) by inhibiting inflammatory pathways such as histone deacetylase (HDAC) and NF-κB (Rekha et al., 2024), whilst promoting the differentiation of regulatory T cells (Tregs) and inhibiting Th17 polarisation, thereby maintaining Treg/Th17 immune homeostasis (Wen et al., 2021). Therefore, based on the research evidence outlined above, it is hypothesised that, in a state of sepsis, reduced SCFA production may lead to impaired intestinal barrier function and an imbalance in immune homeostasis, thereby promoting intestinal inflammation and the development of leaky gut, and further exacerbating the transmission of enteric inflammatory signals to the kidneys.

Both patients with sepsis and animal models have shown that, in sepsis, an imbalance in the gut microbiota disrupts tryptophan metabolism via dual pathways, which may lead to an immune imbalance. Firstly, the reduction in beneficial bacteria leads to decreased production of indole-3-propionic acid (IPA) and indole-3-acetic acid (IAA) (He et al., 2023). This results in insufficient activation of the aryl hydrocarbon receptor (AhR) and disruption of the intestinal barrier, thereby exacerbating systemic inflammation and endotoxin burden (Xu, 2025). Secondly, the kynurenine pathway of tryptophan metabolism is excessively activated, with the key rate-limiting enzyme indoleamine 2,3-dioxygenase being highly expressed. This not only consumes large quantities of tryptophan but also produces a series of metabolites: Among these, toxic substances such as kynurenic acid (KYNA) and quinolinic acid (QA) may upregulate TLR4 expression via unknown mechanisms, thereby exacerbating inflammation (Xu, 2025). On the other hand, depletion of tryptophan inhibits G1-phase T cell proliferation and effector T cell activation, instead promoting the conversion of naive T cells into regulatory T cells, thereby inducing immune imbalance (Lu et al., 2026).

Disorders of choline metabolism may also exacerbate damage to the intestinal barrier: under normal circumstances, the gut microbiota metabolises dietary choline into trimethylamine (TMA), which is subsequently converted by the liver into trimethylamine oxide (TMAO) before being excreted via renal metabolism (Huang and Chen, 2024). Although direct evidence remains limited, existing research suggests that TMAO can activate the intestinal TLR4/MyD88/NF-κB signalling pathway (Li J. et al., 2025) and promote intestinal inflammation; it is therefore hypothesised that sepsis-associated dysbiosis may lead to the accumulation of TMAO, which in turn further impairs the intestinal barrier and amplifies the inflammatory response mediated by the gut-kidney axis.

In summary, sepsis-associated dysbiosis not only alters the microbial composition but also, through metabolic reprogramming processes such as reduced SCFAs, abnormal tryptophan metabolism and TMAO accumulation, synergistically drives intestinal barrier damage, immune dysregulation and persistent inflammatory responses, thereby promoting kidney injury mediated by the gut-kidney axis.

### 2.3 Immune-cell-mediated cross-talk between intestinal and renal inflammation and immunometabolic remodelling

Against a background of intestinal barrier damage and dysbiosis, gut-derived inflammatory signals further promote the activation of the immune system and contribute to the process of renal inflammatory damage. Immune cells, acting as important mediators linking the gut and the kidneys, play a key role in the onset and progression of SA-AKI. Gut-derived bacterial components, inflammatory cytokines and signals of metabolic abnormalities can enter the kidneys via the circulation, inducing the local recruitment and activation of immune cells, thereby promoting the sustained amplification of the inflammatory response.

This immune crosstalk between the gut and kidneys has been further supported by relevant studies on immune interventions (Liu et al., 2025): In a mouse model of sepsis, adoptive transfer of TPP-loaded macrophages (TPA2PyPh-loaded macrophages) demonstrated preferential accumulation at sites of infection, including the peritoneal cavity and kidneys. This treatment not only effectively cleared bacteria from multiple tissues, including the kidneys, but also significantly reduced systemic inflammatory cytokine levels (TNF-α, IL-6). This indirectly indicates that the inflammatory microenvironment within critical organs such as the kidneys was effectively ameliorated. The study further suggests that the regulation of immune cell function may influence the inflammatory state of the gut-kidney axis. Furthermore, Zhang Huali’s team (Tan et al., 2025) discovered that IL1R2 (IL-1 receptor 2) inhibits glycolysis by binding to ENO1 (Enolase 1) in macrophages. This process reduces pyroptosis and inflammatory responses, ultimately protecting mice from organ damage and death induced by sepsis. This mechanism also offers a potential new approach to controlling renal inflammation.

In addition to the direct effects of inflammatory cytokines, gut microbiota-associated metabolites may also influence the inflammatory state of the kidneys by modulating the function of immune cells, thereby contributing to the immune regulatory processes of the gut-kidney axis. SCFAs promote the differentiation of naive T cells into Tregs and enhance Treg proliferation and function by inhibiting HDACs and activating G protein-coupled receptors (GPRs) such as GPR43 (Saadh et al., 2025). When SCFAs decrease, this crucial immunoregulatory function of Tregs is impaired. Moreover, a number of cutting-edge experimental studies and clinical observations have elucidated the role of the gut microbiota and bile acids in immune regulation and metabolic health. In theory, during the progression of sepsis, dysbiosis disrupts the normal microbial conversion of primary bile acids into secondary bile acids. This impairs the activation of key bile acid receptors such as the farnesoid X receptor (FXR) and G protein-coupled bile acid receptor 1 (GPBAR1). This disruption weakens FXR’s anti-inflammatory effects in monocytes/macrophages and obstructs GPBAR1-mediated core immune regulatory functions. These include direct suppression of macrophage production of pro-inflammatory factors such as TNF-α and IL-6, alongside the ability to direct monocyte differentiation towards tolerogenic dendritic cells to induce Tregs and suppress Th1/Th17 responses (Tyagi and Kumar, 2025). The inactivation of these receptors collectively leads to a weakening of anti-inflammatory mechanisms and a relative enhancement of pro-inflammatory responses, which in turn promotes the infiltration of hyperactivated macrophages and dysregulated T-cell subsets into the kidneys, thereby exacerbating the inflammatory response within the kidneys and driving the progression of SA-AKI. However, this hypothesis requires further validation in SA-AKI models to clarify its causal relationship and potential for clinical translation.

Using a model of LPS-stimulated macrophages, Tan et al. demonstrated that the hyper-inflammatory state associated with sepsis can induce metabolic reprogramming in immune cells, causing them to switch to aerobic glycolysis (the Warburg effect) even under conditions of adequate oxygen supply, thereby promoting inflammasome activation and IL-1β production (Tan et al., 2025). This study suggests that immunometabolic abnormalities may further promote renal inflammatory damage by enhancing the activation of inflammatory cells and the release of pro-inflammatory factors. Furthermore, pharmacological or genetic inhibition of ENO1 may reduce GSDMD-mediated pyroptosis by decreasing glycolysis-driven caspase-1 activation, thereby offering a potential avenue for targeted immunometabolic regulation in the treatment of SA-AKI.

Consequently, immune cell infiltration does not occur in isolation within the kidney, but is jointly influenced by gut-derived inflammatory signals, gut microbiota metabolic abnormalities and immune regulatory imbalances; it represents a critical link driving the transition from intestinal inflammation to renal injury.

### 2.4 Metabolite-mediated gut-kidney feedback loops and disease progression

Compared with intestinal barrier damage, dysbiosis and immune dysfunction, metabolite-mediated interactions further constitute a feedback regulatory mechanism within the gut–kidney axis. On the one hand, gut-derived metabolites can directly influence renal inflammation and oxidative stress; on the other hand, impaired renal function leads to reduced metabolite clearance, which in turn further exacerbates gut microbiota dysbiosis.

The accumulation of uraemic toxins and disruption of the intestinal barrier form a vicious cycle, jointly exacerbating renal injury and systemic inflammation. On the one hand, declining renal function impairs the excretion of substances such as indole-3-carbinol sulphate (IS), p-cresol sulphate (p-CS), and TMAO. These toxins further damage renal tissue by inducing oxidative stress, inflammatory responses, and vascular injury (Tsuji et al., 2024; Cedillo-Flores et al., 2025), On the other hand, these uraemic toxins themselves originate from the gut microbiota’s metabolism of dietary components (such as the production of indole/para-cresol precursors from protein metabolism, and TMAO precursors from choline metabolism) (Sun et al., 2021; Huang and Chen, 2024), their accumulation, in turn, can exacerbate gut microbiota dysbiosis and barrier dysfunction, thereby promoting the persistence of abnormalities in the gut-kidney axis.

Moreover, advanced glycation end products (AGEs) can promote the amplification of inflammation and the disruption of the barrier by activating their receptor, RAGE, thereby further exacerbating the imbalance in the gut-kidney axis (Li Y. et al., 2025).

Overall, metabolic abnormalities transform the gut-kidney axis from a unidirectional damage process into a continuously amplifying feedback loop. Renal impairment leads to the accumulation of uremic toxins and abnormal metabolites, which in turn further damage the intestinal barrier, promote dysbiosis and inflammatory responses, ultimately perpetuating the vicious cycle of sepsis-associated gut-kidney axis damage.

## 3 Innovation in diagnostic and assessment strategies

The multi-faceted pathological changes associated with an imbalance in the gut-kidney axis not only drive the onset and progression of SA-AKI, but also reveal potential windows for disease monitoring. However, existing diagnostic markers primarily reflect consequential changes following renal dysfunction and are unable to promptly detect early pathological events such as intestinal barrier disruption, inflammatory activation and metabolic disturbances. Therefore, novel biomarkers developed based on key pathological mechanisms of the gut-kidney axis are expected to enable dynamic monitoring of the SA-AKI disease process and improve capabilities for early diagnosis and risk stratification.

### 3.1 Limitations of traditional diagnostic indicators

As the most commonly used marker of renal function, serum creatinine (SCr) shares recognised limitations with urine output. SCr levels are susceptible to interference from numerous factors including liver function, muscle mass, metabolism, diet, nutritional status, and medication; meanwhile, the accuracy of urine output measurements is easily compromised by diuretic use or measurement errors (Ostermann et al., 2025). Research indicates that relying solely on these traditional indicators proves inadequate for accurately reflecting changes in renal function or enabling early identification of acute kidney injury. Furthermore, regarding gastrointestinal dysfunction induced by sepsis, assessment currently relies primarily on the Acute Gastrointestinal Injury (AGI) grading scheme and the Gastrointestinal Dysfunction Score (GIDS) (Tyszko et al., 2023), with no established biomarkers yet available for clinical application.

### 3.2 The application prospects of novel biomarkers

In light of these limitations, researchers have begun to explore novel biomarkers capable of overcoming the time-window constraints of traditional markers and reflecting the early pathological changes in SA-AKI. Within the framework of the gut-kidney axis mechanism of SA-AKI, relevant biomarkers can be classified into three categories based on their origin and function: ① Enteric biomarkers, reflecting the structural and functional integrity of the intestinal barrier; ② renal injury biomarkers, indicating the kidney’s response to injury; ③ systemic inflammatory and immune markers, acting as a “bridge” between the gut and kidneys, reflecting the activation of the systemic inflammatory response. These are elaborated and summarised in tabular form below (Table 1).

**TABLE 1:** Novel biomarkers for sepsis-associated acute kidney injury.

Biomarker categories	Designation	Source/Function	Pathophysiological significance	Changes in sa-aki	Advantages	Limitations	Association with the gut -Kidney axis /Prospects for application	Levels of evidence
Enteric biomarkers	I-FABP	Mature small intestinal epithelial cells (Piccioni et al., 2024)	Reflecting intestinal mucosal ischaemia or disruption of the barrier function (Piccioni et al., 2024)	Released when intestinal epithelial cells are damaged or undergo apoptosis; levels may be elevated in SA-AKI.	Highly gut-specific; early release with rapid response	Low specificity; levels may also be elevated in conditions such as trauma or abdominal surgery (Coufal et al., 2020)	A direct marker of intestinal epithelial injury, whose elevation may correlate positively with the severity of SA-AKI; combined testing enhances diagnostic accuracy	Mainly preclinical studies; clinical studies are limited
Renal injury biomarkers	CD35-uEV	Originating from podocytes in the glomerulus, present in extracellular vesicles in urine (Li et al., 2025b)	During sepsis, downregulation of CD35 expression on podocyte surfaces or alterations in the vesicular secretion mechanism result in a significant reduction in the number of CD35-uEVs ultimately entering the urine (Li et al., 2025b)	Significant reduction in expression; negatively correlated with AKI severity; facilitates early diagnosis, risk stratification, and prognostic assessment of SA-AKI(Li et al., 2025b)	Specimens are readily obtained non-invasively; the test demonstrates high diagnostic capability, with an AUC reaching 0.89 (Li et al., 2025b)	The relevant research is still in its early stages	These alterations may be associated with renal injury resulting from gut-kidney axis dysfunction, which indirectly leads to podocyte damage	Clinical research
​	NGAL	Neutrophils (predominantly), hepatocytes (during inflammation), renal tubular cells (during injury) (Skrypnyk et al., 2020)	NgAL is released following injury to renal tubular epithelial cells; its levels correlate closely with the severity of systemic inflammatory states, and its elevation may sometimes more directly reflect the inflammatory process itself rather than a single episode of acute kidney injury (Balkrishna et al., 2023)	In the early stages of SA-AKI, serum and urinary NGAL levels both rise significantly. The latter can be detected as early as three hours after AKI onset, reaching peak levels within six hours (Balkrishna et al., 2023; Yang et al., 2023)	Urinary NGAL concentration correlates with the severity of renal infection and may be used for early prediction of SA-AKI(Park et al., 2019)	Nonspecific, elevated levels of which may be observed in systemic infections, inflammatory conditions, and various neonatal complications (Umbro et al., 2016; Yang et al., 2023)	NGAL maintains gut microbiota stability through its antibacterial, immunomodulatory, and gut permeability-modifying functions, thereby reducing bacterial translocation and systemic inflammation, which may confer renal protection (Yang et al., 2023)	Most of these are preclinical studies; there is a lack of large-scale clinical trials
	[TIMP-2] [IGFBP7]	Secretion by renal tubular epithelial cells (Fiorentino et al., 2020)	Elevated levels indicate G1 cell cycle arrest, signalling AKI(Fiorentino et al., 2020)	Elevated urinary [TIMP-2] [IGFBP7]levels indicate an increased risk of developing moderate-to-severe AKI within the next 12 h; a decrease in these markers following fluid resuscitation reflects a reduced risk of SA-AKI progressing to an unfavourable outcome (Fiorentino et al., 2020)	FDA approval, early warning (Di Leo et al., 2018)	Expensive and unable to indicate the cause of the illness	The combined application of the furosemide stress test with [TIMP-2] [IGFBP7] assays enables precise identification of high-risk SA-AKI patients requiring RRT (Palmowski et al., 2024). This strategy offers a highly promising direction for the clinical translation of research on the sepsis-enteric-renal axis	Progressing from clinical trials to the market validation stage
​	Penkid	Stable fragment of the enkephalin precursor protein; may regulate diuresis and urinary sodium excretion (Walczak-Wieteska et al., 2024)	Persistent SA-AKI patients exhibit significantly elevated Penkid levels, which precede increases in Scr(Walczak-Wieteska et al., 2024)	In SA-AKI, Penkid provides a more reliable reflection of true GFR. The PENK-Crea equation based on Penkid demonstrates excellent performance in estimating GFR, matching or surpassing traditional equations (Walczak-Wieteska et al., 2024)	Early prediction and risk alerting; more directly and sensitively reflecting renal filtration function; associated with disease severity and poor prognosis (Ji et al., 2025)	Comparative studies on clinical predictive value are limited (Ji et al., 2025)	Penkid levels are independently associated with SA-AKI severity, renal function deterioration, 28-day mortality, and the severity of multiple organ failure (Walczak-Wieteska et al., 2024)	Clinical research
Systemic inflammatory and immune markers	sTREM-1	An immunoglobulin superfamily receptor on the surface of myeloid cells (neutrophils, monocytes/macrophages) (Balkrishna et al., 2023)	During infectious diseases, sTREM-1 levels in bodily fluid samples increase (Wang et al., 2025)	During SA-AKI, sTREM-1 may be partially produced by local renal cells (such as renal tubular epithelial cells and infiltrating inflammatory cells), leading to significantly elevated levels in both urine and plasma. These levels are markedly higher than in septic patients without AKI, with the former being of particular value for the early diagnosis of SA-AKI(Derive and Gibot, 2011; Su et al., 2015; Wang et al., 2025)	Elevated levels are risk factors for AKI in patients with sepsis. An upward trend in these levels predicts poor prognosis (Su et al., 2015)	Insufficient specificity. Larger scale, multicentre cohort studies and relevant fundamental research should be conducted (Su et al., 2015)	Early warning; risk stratification; the combination of sTREM-1 with clinical scoring systems or other cross-category biomarkers can significantly enhance the diagnostic and prognostic predictive efficacy for SA-AKI(Balanza et al., 2025)	Preclinical research + clinical research
​	Presepsin	Primarily derived from monocytes/macrophages, it is the soluble N-terminal fragment generated by proteolytic cleavage of the CD14 molecule on their membranes (Velissaris et al., 2021)	Elevated levels can be detected within two hours of infection, with their magnitude correlating to the severity of sepsis (Velissaris et al., 2021)	Patients with SA-AKI exhibit elevated presepsin levels, primarily cleared via renal pathways. These levels correlate with the severity of renal impairment and hold value in assessing the severity and prognosis of sepsis (Velissaris et al., 2021; Balkrishna et al., 2023)	High sensitivity and good specificity, enabling rapid detection	The test has not yet been adopted as a routine laboratory procedure. Renal function interference. Existing studies have small sample sizes, limiting the reliability of the results (Velissaris et al., 2021)	Considered a promising therapeutic target in SA-AKI. When combined with other biomarkers, it forms a multi-marker diagnostic strategy (Velissaris et al., 2021)	Clinical research
	SII	SII = Platelet count × Neutrophil/lymphocyte ratio (Kosidło et al., 2023)	Reflects the pathological processes of platelet activation, neutropenia, and lymphocyte depletion occurring during infection and inflammation (De Jager et al., 2010; Morrell et al., 2014)	The short-term mortality risk in SA-AKI patients exhibits a J-shaped relationship: the lowest risk point occurs at an SII level of approximately 760.078 × 10^9^/L (Sun et al., 2024)	Simple, intuitive, economical, and convenient	Unable to reflect the dynamic evolution of the inflammatory state (Zhang et al., 2024), Low specificity	The direct link between SII and the gut-kidney axis remains to be clearly elucidated. However, the systemic inflammatory state it reflects may serve as a bridge connecting gut microbiota dysbiosis, immune imbalance, and renal injury (Sun et al., 2024)	Clinical research

Abbreviations: SA-AKI, sepsis-associated acute kidney injury; I-FABP, intestinal fatty acid binding protein; NGAL, neutrophil gelatinase-associated lipocalin; [TIMP-2] [IGFBP7], [tissue inhibitor of metalloproteinases-2] [insulin-like growth factor-binding protein 7]; SII, systemic inflammatory response index.

The biomarkers currently used for SA-AKI each have their own potential, but all are hampered by key shortcomings such as specificity, feasibility of detection or the level of evidence. Although I-FABP can reflect intestinal barrier damage in real time, it is susceptible to interference from confounding factors such as intestinal ischaemia and infection, making it difficult to use for independent diagnosis; NGAL has high sensitivity, but inflammatory states can easily lead to false positives, and its detection window and operational costs limit its use as an auxiliary diagnostic tool; CD35-uEV performs well in prognostic assessment; however, the single exosome detection process is complex and expensive, and its specificity in other AKI subtypes remains to be clarified; the combined markers [TIMP-2]·[IGFBP7] demonstrate good predictive performance, but consistency is lacking in non-septic AKI, there is no standardised clinical threshold, and evidence to guide specific treatment modifications is absent; Penkid is currently based solely on observational studies, with no randomised controlled validation; neither the criteria for dynamic monitoring nor the underlying biological mechanisms are clear; sTREM-1 remains in the exploratory phase and requires large-scale prospective trials to substantiate its value; although Presepsin is detected earlier than traditional markers, its levels are significantly influenced by renal function, leading to a high risk of false positives in AKI, and there is a lack of specific evidence for SA-AKI; SII has shown predictive potential in observational cohorts, but the evidence grade is only IIb–III, and there remains a lack of understanding regarding its mechanism of action and prospective validation. Overall, the clinical translation of these biomarkers collectively faces obstacles such as insufficient multicentre validation, a lack of standardisation in testing, and inconsistent thresholds. In the future, stratified validation and mechanistic analysis targeting their respective specific shortcomings will be required to clarify their precise role in clinical decision-making.

### 3.3 Multi-omics technology and integrated analysis

The current research paradigm has shifted from observing microbial community composition towards employing multi-omics technologies to delve into the functional interactome, thereby elucidating protective mechanisms at the molecular level. This transition is driving the emergence of more targeted microbiome therapies.

Xu et al., by establishing a mouse model of LPS-induced SA-AKI and combining proteomics and metabolomics analyses, revealed the central role of mitochondrial dysfunction and metabolic abnormalities in the disease. They identified 10 pivotal proteins associated with the mitochondrial respiratory chain and ribosomes. Providing a basis for potential therapeutic targets. Furthermore, the study found an accumulation of polyamine degradation products (such as N-acetylspermine) and a significant enrichment of the nicotinamide metabolic pathway, suggesting that nicotinamide (NAM) supplementation may exert a protective effect by maintaining NAD + levels and counteracting oxidative stress, thereby representing a potential therapeutic strategy (Xu et al., 2023).

By systematically integrating multimodal data from mouse models and clinical samples, Huang et al. comprehensively elucidated the metabolic changes characteristic of the early stages of SA-AKI, successfully identified five core metabolites and developed the diagnostic model IC3, which achieved an area under the curve of 0.90 (Huang et al., 2025). The potential clinical utility of this diagnostic model lies in its ability to enable early and accurate screening, risk assessment and determination of disease severity for SA-AKI through the analysis of key metabolites in the blood, thereby helping to guide timely intervention and treatment, and consequently reducing patient mortality and the incidence of complications. However, a limitation is that the current model validation is restricted to a relatively small clinical sample; external validation is required in a broader and more diverse clinical population to ensure its stability and generalisability. Furthermore, the model relies on high-precision metabolic testing technology, which may be influenced by equipment and laboratory conditions, thereby limiting its potential for widespread and immediate application.

### 3.4 Advances in imaging and functional assessment

Research by Stephen et al. utilising a mouse model of sepsis revealed that sepsis can directly induce significant renal dysfunction via inflammatory signalling pathways. Nuclear magnetic resonance metabolomics analysis of serum and urine indicated that core metabolic cofactors and signalling pathways associated with mitochondrial function play a pivotal mediating role in this process. Further investigations demonstrated that gene expression of multiple enzymes within key cellular energy supply pathways was suppressed. Moreover, heightened inflammatory levels were confirmed to predict diminished renal cortical fatty acid oxidation capacity and renal dysfunction in septic mice (Standage et al., 2021). These studies identified branched-chain amino acid metabolism, fatty acid oxidation, and the *de novo* synthesis pathway of nicotinamide adenine dinucleotide (NAD^+^) as potential targets for future metabolic interventions in SA-AKI.

It is noteworthy that these renal metabolic disorders are closely associated with intestinal function. The gut-kidney axis mechanism suggests that sepsis-induced intestinal barrier damage, dysbiosis, and microcirculatory dysfunction may represent key factors driving distal renal injury. Therefore, concurrent assessment of intestinal and renal function in septic patients is paramount, with bedside ultrasound demonstrating significant potential in this domain: single transverse scanning of the gastric antrum enables measurement of gastric antral contraction amplitude (ACA), gastric antral contraction frequency (ACF), gastric antral motility index (MI), and gastric emptying time (GET) to evaluate gastric emptying function. Research has further revealed that patients with sepsis complicated by AGI exhibit a significantly increased gastric antrum cross-sectional area, demonstrating superior predictive value compared to small bowel wall thickness (Li et al., 2021). In renal assessment, Liu H. et al. (2023) utilised Doppler ultrasound combined with contrast-enhanced ultrasound (CEUS) to observe that patients with SA-AKI exhibited reduced macrovascular renal blood flow alongside prolonged microcirculatory time parameters, with these alterations independent of cardiac output. The study further indicated that when the renal artery resistance index (RI) ≥ 0.695 or the CEUS peak time to peak (TTP) ≥ 28.4 s, vigilance is warranted regarding the risk of patients progressing to severe AKI. Moreover, assessment of intestinal microcirculation yields crucial insights. Utilising emerging photoacoustic endoscopy technology (Chen et al., 2023), researchers have observed significant alterations in rectal vascular architecture and haemoglobin saturation within animal models of sepsis. This provides a powerful visualisation tool for investigating the mechanisms underlying intestinal microcirculatory dysfunction.

## 4 Therapeutic strategies and prospects for intervention

### 4.1 Regulation of the gut microbiome

Gut microbiota play a pivotal role in maintaining intestinal microecological homeostasis and promoting the differentiation and maturation of immune cells through nutritional metabolism, thereby exerting a profound influence on host immunity.

Research has shown that specific probiotic strains can increase levels of bile acid metabolites by modulating the composition of the gut microbiota, thereby activating key receptors such as FXR and GPBAR1, which are crucial for maintaining the integrity of the intestinal barrier and immune balance (Tyagi and Kumar, 2025). Furthermore, a study by Gu et al. found that levels of *Candida* albicans were significantly reduced in patients with bacterial sepsis, and that the culture supernatant of this fungus could reduce bacterial load and alleviate symptoms of sepsis in animals (mice and pigs); Integrated metabolomics and fungal genetic engineering have confirmed that phenylpyruvic acid (PPA), produced by *Candida* albicans, enhances the bactericidal activity of macrophages and reduces organ damage during sepsis (Gu et al., 2023). Both of these findings are currently at the preclinical and early translational research stages and require further validation.

At the level of clinical validation, the Winner cohort study demonstrated that early exposure to anti-anaerobic antibiotics is independently associated with a 61 percent increased risk of SA-AKI, whilst increased gut bacterial density and the enrichment of Enterobacteriaceae and Lachnospiraceae can predict the subsequent onset of AKI (Winner et al., 2025). Furthermore, the findings of Cedillo-Flores et al. indicate that interventions involving probiotics, prebiotics and synbiotics can effectively reduce levels of uraemic toxins such as indophenol sulphate and p-cresol sulphate (Cedillo-Flores et al., 2025).

### 4.2 Immune and inflammatory targeted therapies

The functional regulation of macrophages constitutes a central theme within this field. On the one hand, researchers are endeavouring to reprogramme macrophage functions. For instance, the research team led by Liu developed the lipid droplet-enriched luminescent molecule TPA2PyPh. By loading this molecule into macrophages and performing adoptive transfer, they successfully cleared invading bacteria in septic mice and alleviated the immunosuppressive microenvironment within the body, offering a novel approach for treating advanced sepsis accompanied by immunosuppression (Liu et al., 2025) --this research is at the preclinical and early translational stages. On the other hand, it is equally important to suppress its excessive activation. Research reveals that defects in the mitochondrial antiviral signalling protein (MAVS) release inhibition of the TLR4 pathway, leading to uncontrolled M1-type polarisation of macrophages. This is accompanied by excessive mitochondrial ROS production, ultimately exacerbating renal injury (Tran et al., 2024). This suggests that targeting the MAVS signalling pathway to regulate redox homeostasis may represent an effective strategy for mitigating inflammatory damage in SA-AKI; however, this work is currently at the preclinical stage.

**FIGURE 2:**
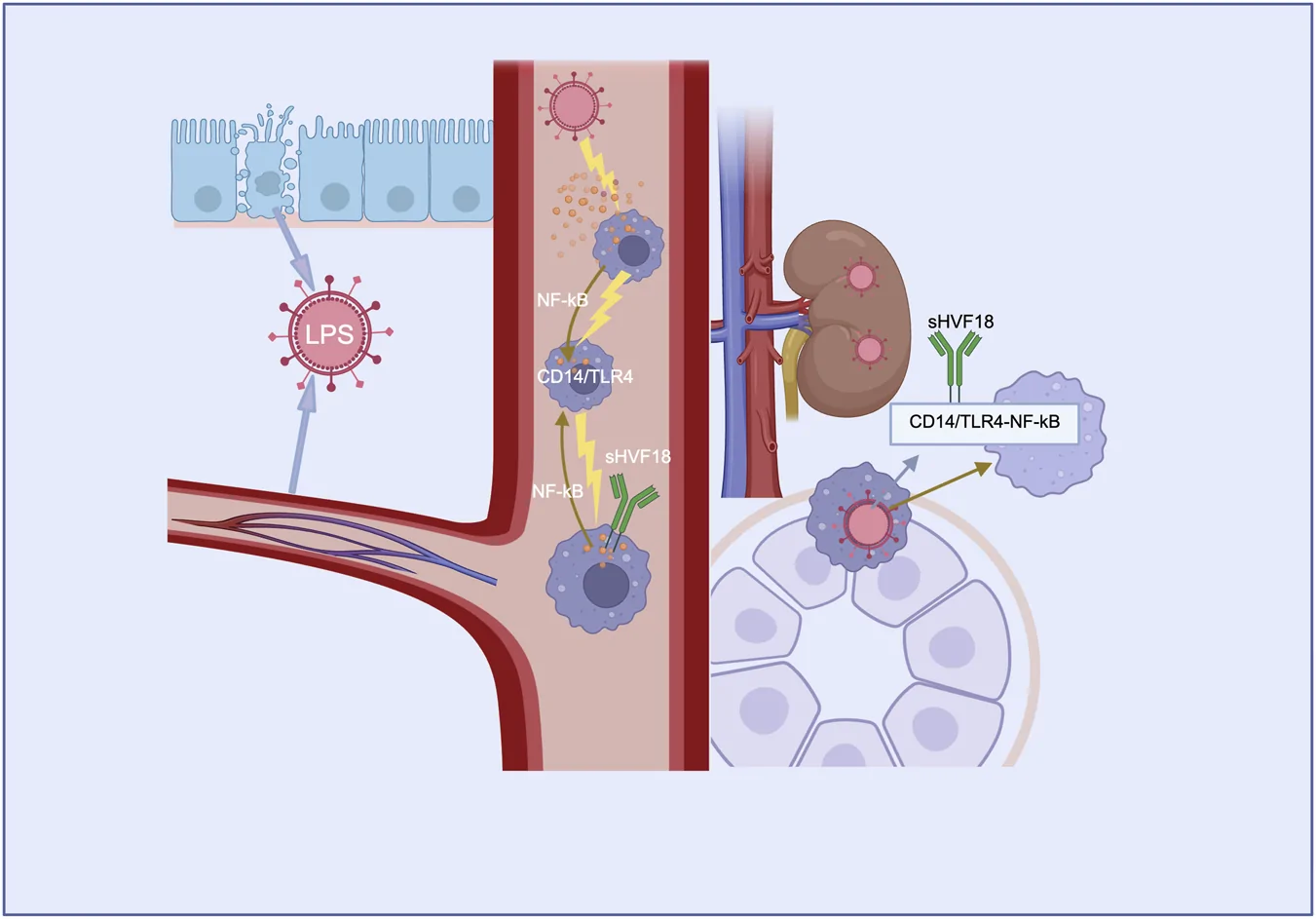
Displaced enteric LPS promotes the activation of intrinsic renal cells and infiltrating immune cells by activating the CD14-TLR4-NF-κB signalling pathway, thereby inducing the release of pro-inflammatory cytokines and exacerbating damage to glomerular and tubular epithelial cells. Preclinical studies suggest that the microbiota-derived peptide sHVF18 can alleviate sepsis-associated acute kidney injury by neutralising endotoxins and targeting the immune receptor CD14, thereby regulating inflammatory signalling at an upstream level; however, its therapeutic efficacy, safety and clinical translational value require further validation.

The engineered peptide sHVF18 can neutralise endotoxins and target the immune receptor CD14 in animal models, thereby inhibiting systemic inflammation at an upstream level to alleviate SA-AKI(Petruk et al., 2025) (Figure 2); Similarly, miR-6722–3p alleviates renal inflammation and apoptosis by inhibiting the pro-inflammatory factor CHI3L1 and blocking the activation of the TLR4/NF-κB signalling pathway (Zhang et al., 2025). However, both are currently confined to the preclinical stage, and their efficacy and safety require validation through multiple rounds of clinical trials before their therapeutic value can be confirmed and they can progress to clinical application.

### 4.3 The potential value of traditional Chinese medicine

In recent years, attention has been drawn to the potential value of plant-derived active compounds in the prevention and treatment of SA-AKI, providing a promising avenue for clinical translation.

With regard to plant-derived compounds, Zhu et al. observed in a CLP-induced sepsis model in young mice that oral administration of baicalin significantly reduced blood urea nitrogen and serum creatinine levels, this protective effect was associated with the inhibition of apoptosis in renal tubular epithelial cells (Zhu et al., 2016). Curcumin has also been shown to alleviate inflammatory responses and apoptosis in the CLP model, a mechanism involving the inhibiting of the NF-κB and JAK2/STAT3 signalling pathways (Balkrishna et al., 2023). Resveratrol has been reported to alleviate endoplasmic reticulum stress and inflammatory damage by activating the Nrf2 antioxidant pathway and inhibiting the NF-κB pathway (Wang et al., 2018).

With regard to traditional Chinese herbal formulations, some studies have begun to explore indirect protective mechanisms based on the gut microbiota. Research indicates that the Liangxue Huoxue Formula can, by improving the species diversity of the gut microbiota and reducing the permeability of intestinal barrier in a mouse model of sepsis, thereby inhibit the activation of the NLRP3/caspase-1/GSDMD signalling pathway in renal tissue and the expression of inflammatory cytokines IL-1β and IL-18, thus alleviating renal histopathological damage (Zhou et al., 2023). Using spatial metabolomics techniques, Qiao et al. found that Si Ni Tang, through its various components that enter the bloodstream and cross the blood-brain barrier, can systematically regulate metabolic dysregulation of the hypothalamic-pituitary-adrenal axis (HPA) axis during sepsis. This involves multiple pathways, including glycerophospholipid metabolism, fatty acid oxidation, tryptophan metabolism, and fatty acid amide metabolism, thereby suppressing excessive inflammatory responses and protecting organ function (Qiao et al., 2025).

It should be noted that the aforementioned studies were all preclinical animal experiments, and it remains to be verified whether their conclusions can be extrapolated to human patients with SA-AKI. To date, evidence regarding the clinical application of plant-derived active compounds or traditional Chinese medicine formulations in the treatment of SA-AKI is extremely limited, and there is a lack of prospective randomised controlled trials (RCTs). Future research should build upon animal studies to clarify the pharmacokinetic characteristics of the active compounds and evaluate their efficacy and safety through rigorously designed clinical trials, thereby advancing the translation of research in this field from the laboratory to clinical practice.

### 4.4 Other emerging therapeutic approaches

Research indicates that the core mechanism by which vagus nerve electrical stimulation protects against acute kidney injury in sepsis lies in activating the cholinergic anti-inflammatory pathway. This pathway exerts renal protective effects by suppressing systemic inflammation, mitigating tubular injury, and regulating immune homeostasis (Shi et al., 2022; Li et al., 2024). This mechanism is consistent with the concept of the “brain-gut-kidney axis” proposed by Yang et al., in 2018, wherein the gut microbiota communicates with the brain via three parallel and interacting pathways: neural, immune, and metabolic (Yang et al., 2018). This provides novel strategies for neuroimmunological interventions in SA-AKI.

**FIGURE 3:**
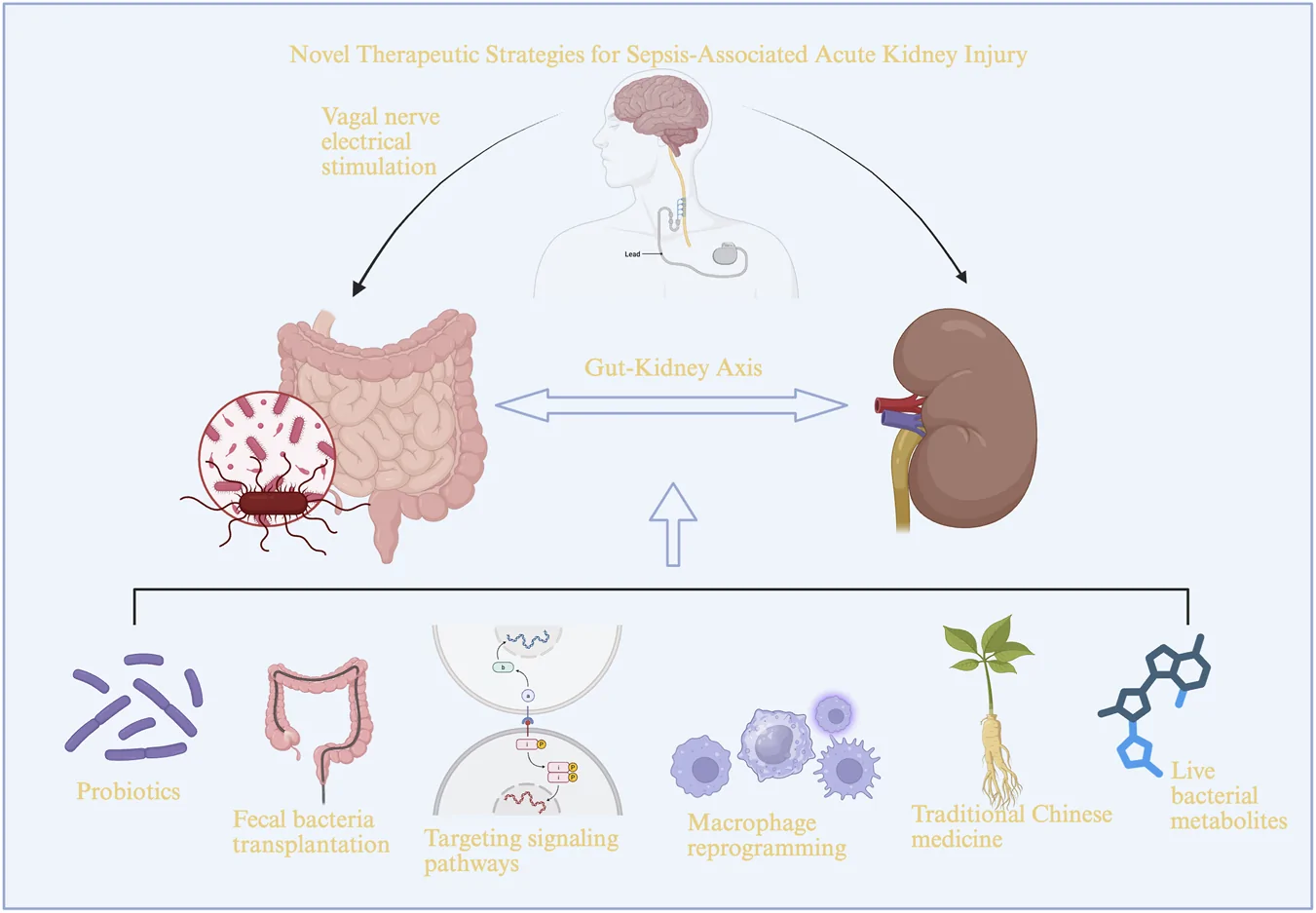
This figure summarises potential intervention strategies for SA-AKI based on regulation via the brain–gut–kidney axis and the gut–kidney axis. Vagus nerve stimulation can influence the inflammatory response by modulating neuro-immune interactions and cholinergic anti-inflammatory pathways; intervention strategies targeting the gut–kidney axis include probiotics, faecal microbiota transplantation, macrophage reprogramming, traditional Chinese medicine, targeted therapy of inflammatory signalling pathways, and microbiota-derived metabolites. These strategies aim to mitigate the transmission of enteric inflammatory signals to the kidneys and improve renal injury by modulating the gut microbiota, immune and inflammatory responses, and metabolic homeostasis. However, most of these strategies are currently at the preclinical or early translational stage, and their efficacy, safety and clinical utility in patients with SA-AKI require further validation.

Xie et al. conducted metabolomic analyses and sterilised mouse validation experiments demonstrating that viable Akkermansia mucilaginosa (AKK) bacteria produce a novel tripeptide Arg-Lys-His (RKH), which exerts protective effects against sepsis-induced organ damage and mortality. Histological analysis revealed that RKH-treated mice exhibited reduced inflammation and injury in multiple organs (including lungs, liver, kidneys, and heart) following CLP surgery; Plasma biochemical assays revealed significantly lower levels of alanine aminotransferase, aspartate aminotransferase, creatine kinase, creatinine, and urea in RKH-treated septic mice compared to controls. Furthermore, reduced mRNA levels of inflammatory cytokines were observed in the lungs, liver, kidneys, and hearts of RKH-treated septic mice (Xie et al., 2024). RKH holds promise as a novel potential therapeutic approach for combating lethal sepsis and alleviating SA-AKI (Figure 3). Both of the above studies are at the preclinical stage.

## 5 Summary and outlook

### 5.1 Current status, challenges and key scientific issues in research on the gut-kidney axis

The onset and progression of SA-AKI are increasingly recognised as a multi-organ interactive process driven by a combination of intestinal barrier damage, dysbiosis, immune abnormalities and metabolic disturbances, rather than simply local renal damage. In recent years, the gut-kidney axis theory has provided a new perspective for understanding the complex pathophysiological processes of SA-AKI. Relevant studies have shown that sepsis-induced disruption of the intestinal barrier facilitates the entry of bacterial components, inflammatory mediators and microbiota-associated metabolites into the circulation, thereby further amplifying the systemic inflammatory response and exacerbating renal injury; simultaneously, metabolic abnormalities resulting from renal dysfunction can in turn affect the homeostasis of the gut microbiota, creating a persistent vicious cycle.

However, research on the gut-kidney axis currently faces numerous challenges. Firstly, existing studies have primarily revealed correlations between intestinal abnormalities and renal injury; yet, there remains a lack of sufficient causal evidence to demonstrate whether intestinal alterations directly drive the onset and progression of SA-AKI. Secondly, the gut-kidney axis may be dynamically regulated by multiple factors, including disease stage, type of infection and host status, and the key regulatory mechanisms at different stages have not yet been fully elucidated. Furthermore, current evidence is derived primarily from animal models and basic research; however, differences between experimental models and clinical sepsis patients—in terms of infection source, immune status and microbial composition—limit the translation of research findings into clinical practice. Therefore, future research should integrate clinical cohorts, multi-omics techniques and dynamic monitoring strategies to further elucidate the key regulatory nodes of the gut-kidney axis and their characteristics at different disease stages, thereby advancing the field from ‘association discovery’ to ‘causal validation’.

### 5.2 From mechanistic discoveries to biomarker development: opportunities and limitations

Given the multi-level pathological changes associated with abnormalities in the gut-kidney axis, a variety of candidate biomarkers have been utilised for the prediction of SA-AKI risk, early diagnosis and prognostic assessment. However, most of these markers currently suffer from issues such as insufficient specificity, susceptibility to inflammatory states and organ function, and a lack of large-scale clinical validation.

It should be emphasised that SA-AKI-related markers do not merely reflect damage to a single organ, but rather represent the combined result of multiple pathways, including disruption of the intestinal barrier, inflammatory activation, dysbiosis and renal injury. Therefore, future biomarker research should not be limited to identifying single indicators, but should instead establish a multidimensional dynamic evaluation system based on the pathophysiological mechanisms of the gut-kidney axis, integrating information on microbiota characteristics, metabolic abnormalities, immune status and renal injury. At the same time, the relationship between candidate biomarkers and disease mechanisms as well as treatment response needs to be further clarified in order to enhance their clinical utility.

### 5.3 From mechanistic targets to precision interventions: future directions for clinical translation

Although intervention strategies targeting the gut-kidney axis—including microbiota modulation, barrier protection, inflammation regulation and metabolic interventions—have demonstrated potential therapeutic value in preclinical studies, most of these strategies remain at an exploratory stage, and their safety, efficacy and clinical applicability require further validation. Previous research into the treatment of sepsis has shown that, due to the highly heterogeneous nature of the disease, single-target interventions often fail to improve overall clinical outcomes. Consequently, future treatment strategies for SA-AKI need to shift from traditional single-target treatment models towards precision intervention models tailored to patient phenotype, disease stage and key pathophysiological mechanisms. For example, selecting appropriate intervention strategies based on the extent of intestinal barrier damage, microbiota characteristics and metabolic status in individual patients may represent a key direction for improving treatment outcomes.

Overall, further breakthroughs in research on the gut-kidney axis will depend on a shift from ‘identifying associations’ to ‘validating causality, establishing dynamic evaluation systems and guiding precision interventions’. By combining multi-omics technologies, patient stratification strategies and clinical validation studies, it is hoped that treatment strategies related to the gut-kidney axis will gradually progress from experimental research to clinical application.
